# Geochemistry shapes microbial diversity and selected functional traits in flowback and produced waters from hydraulically fractured formations

**DOI:** 10.1093/femsec/fiag070

**Published:** 2026-06-30

**Authors:** Ya Deng, Mikayla A Borton, Camilla L Nesbø, Malcolm D Forster, Kurt O Konhauser, Murray K Gingras, Greg G Goss, Kelly C Wrighton, Brian D Lanoil, Cheng Zhong, Daniel S Alessi

**Affiliations:** College of Chemistry and Chemical Engineering, Southwest Petroleum University, Chengdu, Sichuan, 610500, China; College of Agricultural Science, Colorado State University, Fort Collins, Colorado, 80523, United States; Department of Biological Sciences, University of Alberta, Edmonton, Alberta, T6G 2E9, Canada; Department of Biological Sciences, University of Alberta, Edmonton, Alberta, T6G 2E9, Canada; Department of Earth and Atmospheric Sciences, University of Alberta, Edmonton, Alberta, T6G 2E3, Canada; Department of Earth and Atmospheric Sciences, University of Alberta, Edmonton, Alberta, T6G 2E3, Canada; Department of Biological Sciences, University of Alberta, Edmonton, Alberta, T6G 2E9, Canada; College of Agricultural Science, Colorado State University, Fort Collins, Colorado, 80523, United States; Department of Biological Sciences, University of Alberta, Edmonton, Alberta, T6G 2E9, Canada; College of Chemistry and Chemical Engineering, Southwest Petroleum University, Chengdu, Sichuan, 610500, China; Department of Earth and Atmospheric Sciences, University of Alberta, Edmonton, Alberta, T6G 2E3, Canada; Department of Earth and Atmospheric Sciences, University of Alberta, Edmonton, Alberta, T6G 2E3, Canada

**Keywords:** unconventional oil and gas, hydraulic fracturing, flowback and produced water, microbial ecosystems, geology-engineering integration

## Abstract

Microbial communities inhabiting hydraulically fractured subsurface waters are increasingly recognized as important components of unconventional oil and gas systems because they can influence water quality, infrastructure integrity, and biogeochemical processes during flowback and production. However, a quantitative cross-basin understanding of their taxonomic diversity, ecological organization, and potential functional variation remains limited. In this study, we analyzed 16S rRNA gene amplicons, metagenomes, and geochemical data from flowback and produced water from the Sichuan Basin, China, and conducted a quantitative comparison to data previously reported from the same basin and hydraulic fracturing regions in North America. Our findings revealed strong co-occurrence patterns among fermentative, sulfidogenic, and methanogenic micro-organisms, which emerged as core members of microbial communities across all fractured subsurface environments. Notably, microbial diversity and selected metabolic traits differed across basins in the low-salinity systems of China, whereas high-salinity basins in North America exhibited reduced diversity and more constrained metabolic capabilities. These differences are consistent with salinity acting as an important ecological filter across the analyzed basins. Our results indicate that basin-specific geochemical context, particularly salinity, is closely associated with cross-basin differences in microbial diversity, community composition, and selected metabolic traits in fractured subsurface waters. These findings support the value of integrating geological, geochemical, and microbiological information when interpreting microbial risks and water-management strategies in hydraulic fracturing systems.

## Teaser

Microbial diversity in flowback and produced water is driven by salinity, but core metabolic groups remain consistent across sites.

## Introduction

Horizontal drilling combined with multiple-stage hydraulic fracturing (HF) has enabled unconventional oil and gas (UOG) extraction from low-permeability strata (Zhong et al. 2021a,b). During HF, large volumes of water are injected per well into target geological formations at depths of typically several kilometers, and some laterals may now be stimulated with over 30 million gallons (>100 000 m3) of fluids. Injected fluid combined with formation brine returns to the surface as flowback and produced water (FPW) after the HF operation is completed (Kondash et al. 2018, Zolfaghari et al. 2024). These water cycles interact intensely with the petroleum system, including key features such as source rocks and depth (Table 1). Injected fluid combined with formation brine returns to the surface as FPW after the HF operation is completed (Kondash et al. 2018, Zolfaghari et al. 2024). These water cycles interact intensely with the petroleum system, including key features such as source rocks, thermal maturity, and depth. Table 1 summarizes the analyzed basins and formations and provides geological context for interpreting cross-basin geochemical and microbiological differences.

**Table 1:** Geological and petroleum-system context of the analyzed basins and formations.

Basin/region	Country	Formation/play	Age	Depth (m)	Depositional environment	Lithology	Dominant hydrocarbon type (literature-based)
Sichuan Basin	China	Wufeng–Longmaxi Formation	Ordovician to Silurian	2000–4500	Deepwater shelf	Black siliceous shale and carbonaceous shale, interbedded with thin laminae of siltstone	Dry gas, methane-dominated
Appalachian Basin	U.S.	Marcellus	Devonian	1000–2500	Deep-water basin	Black siliceous shale	Dry gas to liquids-rich / gas-condensate, depending on basin position
Appalachian Basin	U.S.	Utica	Ordovician	500–4500	Deep marine foreland basin	Interbedded calcareous mudstone/shale and marlstone, with local thin limestone interlayers	Oil-rich to wet gas and dry gas across the play
Western Canada Sedimentary Basin	Canada	Duvernay	Devonian	2500–4000	Deepwater shelf	Black and dark brown marlstone, lime mudstone, and mudstone/shale	Oil, condensate/NGL-rich, and gas-prone intervals across the play

Diverse microbial assemblages have been identified in hydraulic fracturing waters from shale gas and shale oil systems in the U.S. (Mouser et al. 2016, Wang et al. 2019), Canada (Zhong et al. 2019), and China (Zhang et al. 2017, 2020). Previous studies have shown that these communities commonly include fermentative bacteria, halophiles, sulfidogenic micro-organisms, and methanogens, and that their composition may shift from injected fluids to later-stage flowback and produced waters (Cluff et al. 2014, Daly et al. 2016, Borton et al. 2018b). Micro-organisms in these systems can utilize both compounds introduced during hydraulic fracturing and substrates derived from the formation. HF fluids typically contain water, proppant, and chemical additives, whereas fractured formations may supply dissolved organic carbon and hydrocarbons as carbon and energy sources, together with sulfur-bearing compounds that may serve as additional electron donors or acceptors (e.g. Daly et al. 2016, Booker et al. 2017, Borton et al. 2018a, Lipus et al. 2017, Borton et al. 2018b, Booker et al. 2019, Evans et al. 2019). Such microbial activity has been linked to biocorrosion, gas souring, reservoir fouling, and challenges in FPW storage, treatment, and reuse, as well as impacts associated with surface releases of FPW (e.g. Daly et al. 2016, Booker et al. 2017 , Nixon et al. 2017, Mumford et al. 2018, Zhong et al. 2020, Tang et al. 2021, Akob et al. 2021, Zhong et al. 2022). However, most previous studies have focused on individual basins, and direct cross-basin comparisons integrating microbial composition with geochemical context remain limited.

In this study, we generated new 16S rRNA gene amplicon, metagenomic, and geochemical data from FPW samples from the Sichuan Basin, China, and reanalyzed published sequence and geochemical datasets from the Sichuan Basin and several hydraulically fractured basins in North America, including the Marcellus, Utica, Niobrara, Bakken/Three Forks, Antrim, Barnett, and Duvernay. Detailed sample numbers, data types, and accession information are summarized in Table S1. Our objective was to compare microbial community composition, diversity, and selected functional potential across basins and to evaluate how these broad patterns relate to geochemical context, particularly salinity. Because this comparison integrates datasets generated using different sequencing approaches, we interpret the results primarily as broad cross-basin ecological patterns in flowback and produced waters from hydraulically fractured formations.

## Materials and methods

### Sampling

Samples were collected from five wells completed in 2018 in the Wufeng–Longmaxi Formation, Sichuan Basin, China. The sampled wells were located in the Weiyuan and Zhaotong shale gas areas within the Sichuan Basin. The five sampled wells included three wells from the Weiyuan shale-gas area (WY-1, WY-2, and WY-3) and two wells from the Zhaotong shale-gas area (ZT-1 and ZT-2). The eight FPW samples comprised one sample from WY-1, four samples from WY-2, one sample from WY-3, one sample from ZT-1, and one sample from ZT-2; one input-water sample from the Weiyuan sampling campaign was also included. A total of eight FPW samples and one input-water sample were collected from gas-fluid separators while produced fluids were entering the surface collection tanks. Samples were collected between 75 and 156 days after the onset of flowback and were used for 16S rRNA gene amplicon sequencing; two of these FPW samples were additionally selected for shotgun metagenomic sequencing (Table 2). No shut-in period occurred between hydraulic fracturing completion and the onset of production for the sampled wells; this information is provided to indicate that the collected fluids represent continuous post-fracturing flowback/production rather than fluids influenced by a prolonged shut-in interval. Fluids were collected in 500 ml sterile polypropylene containers without headspace and transported at 4°C to Southwest Petroleum University, Chengdu, China, within 3 days of collection. The fluid samples were filtered through sterile hydrophilic polypropylene membranes (0.22 µm pore size) and stored at −20°C until DNA extraction.

**Table 2:** Overview of sequence datasets included in the comparative analyses and their analytical use.

Basin/region	Formation/play or shale-gas area	Sample type	Sequence data and sample number	No. of wells	Flowback time (day)	Reference
Sichuan Basin	Wufeng–Longmaxi Formation; Weiyuan and Zhaotong shale-gas areas	FPW	8 16S rRNA gene amplicons; 2 metagenomes	5	75–156	This study
Sichuan Basin	Wufeng–Longmaxi Formation	FPW	2 published metagenomes	Not specified in this table	210, 480	Zhang et al. 2020
Sichuan Basin	Wufeng–Longmaxi Formation; Weiyuan shale-gas area	Input water	1 16S rRNA gene amplicons	1 sampling campaign	–	This study
Duvernay	Duvernay Formation	FPW + input	8 FPW and 2 input-water 16S rRNA gene amplicons	2	0.04–18	Zhong et al. 2019
Marcellus	Marcellus Formation	FPW + input	34 FPW 16S rRNA gene extracts; 16 FPW metagenomes; 1 input-water 16S rRNA gene extract	See source datasets	7–488	Daly et al. 2016, Borton et al. 2018b
Utica	Utica Formation	FPW + input	20 FPW 16S rRNA gene extracts; 2 FPW metagenomes; 5 input-water metagenomes	See source datasets	9–302	Borton et al. 2018b, related public datasets
Niobrara	Niobrara Formation	FPW	1 16S rRNA gene amplicons	1	140	Wang et al. 2019
Bakken/Three Forks	Bakken Formation and Three Forks Formation	FPW	4 16S rRNA gene amplicons	4	8–9	Wang et al. 2019
Antrim	Antrim Shale	FPW	16S rRNA gene amplicon datasets from 3 wells	3	–	Wuchter et al. 2013
Barnett	Barnett Shale	FPW	16S rRNA gene amplicon datasets from 2 wells	2	–	Davis et al. 2012

### Chemical analyses

Chemical analyses of FPW and input water samples from the Sichuan Basin were conducted at the Southwest Petroleum University in Chengdu, China. following standard protocols described in the Supplementary Information (SI). pH and total dissolved solids (TDS) were measured followed standard protocols. The chemical oxygen demand (COD) of water samples was measured by the dichromate method using a spectrophotometer (COD-571–1, INESA, Shanghai, China). The concentrations of cations including Ca^2+^, K^+^, Na^+^, Mg^2+^, and Sr^2+^ were analyzed using an atomic absorption spectrophotometer (AA-7020, EWAI, Beijing, China), and the concentrations of the anions F^-^, SO_4_^2-^, NO_3_^-^, and Cl^-^ were measured using ion chromatography (883, Metrohm, Herisau, Switzerland) following standard protocols. Additional descriptions of geochemical analyses are presented in the Supplementary Information (SI) section.

### DNA extraction, PCR, and 16S rRNA gene amplicon sequencing

Total DNA from eight FPW samples and one input water sample was extracted using the FastDNA™ SPIN Kit for Soil following the protocol of a previous study (Zhong et al. 2019). 16S rRNA genes were amplified targeting V4 regions (341F-806R primers, 5′-CCTAYGGGRBGCASCAG-3′ and 5′-GGACTACNNGGGTATCTAAT-3′) (Yu et al. 2005). The PCR amplicons were sent to a Novogene for Illumina HiSeq sequencing (Illumina, California, USA).

### Shotgun metagenomic sequencing, genome-assembly, annotation, and construction and annotation for the metagenome-assembled genomes

Shotgun metagenomic sequencing libraries with a 350 bp insert size were constructed from the total DNA for FPW samples from the Weiyuan shale gas play collected on days 80 and 156 of one well flowback using the NEBNext® Ultra^TM^ DNA Library Prep Kit (New England Biolabs, Massachusetts, USA) following the standard protocol provided by Illumina. After paired-end sequencing on an Illumina HiSeq2500 platform (Illumina, California, USA), 5.96 and 6.27 Gb of Illumina reads with total lengths of 40 698 462 and 42 775 962 bp, respectively, were generated from each sample.

The raw data were trimmed and quality-checked (SI). Trimmed reads from the two newly generated Sichuan Basin metagenomes were assembled using both MEGAHIT v1.1.2 (Li et al. 2015) and MetaSPAdes (Nurk et al. 2017). Read mapping to assembled contigs was performed using BBMap v37.24 for MEGAHIT-assembled contigs and Bowtie v1.1.2 (Langmead and Salzberg 2012) for MetaSPAdes-assembled contigs. Contigs >2500 bp from each assembly were binned using MaxBin v2.2.7 (Wu et al. 2014) and MetaBAT v1.7 (Kang et al. 2015). Genome bins from all four assembly-binning combinations (MEGAHIT-MaxBin, MEGAHIT-MetaBAT, MetaSPAdes-MaxBin, and MetaSPAdes-MetaBAT) were pooled and dereplicated with dRep v2.3.2 (Olm et al. 2017). Dereplicated bins were assessed for quality using CheckM v1.1.0 (Parks et al. 2015) and assigned to taxonomy using GTDB-Tk v0.2.2 (Chaumeil et al. 2020). MAGs with >90% completeness, <5% contamination, and <200 contigs were selected for further manual refinement (for further details, see SI). MEGAHIT v1.1.2 was retained as part of the local assembly workflow to maintain consistency with the original analysis and downstream refinement of the newly generated Sichuan Basin datasets. The two Sichuan Basin metagenomes are available in IMG under accession numbers 3 300 031 260 and 3 300 031 485, and the corresponding high-quality MAGs are available under GOLD Study Gs0135899.

### Meta-analyses

#### Data sources for meta-analyses of sequences and geochemistry

To understand the microbial communities in FPW at broader scales, we conducted meta-analyses of sequences and geochemistry data. Previously published datasets included samples from the Sichuan Basin, China, and from hydraulically fractured UOG basins in North America. The North American comparison datasets included the Marcellus and Utica (Appalachian Basin, U.S.), Niobrara (Denver-Julesburg Basin, U.S.), Bakken/Three Forks (Williston Basin, U.S.), Barnett (Texas, U.S.), Antrim (Michigan, U.S.), and Duvernay (Western Canada Sedimentary Basin, Canada). Sequencing data were obtained from the NCBI and IMG public databases. An overview of the included datasets, sample numbers, and their analytical use in this study is provided in Table 2, whereas accession-level details are listed in Table S1. For sequence-based comparisons, the compiled dataset included two published metagenomes from the Sichuan Basin (Zhang et al. 2020), together with North American FPW datasets comprising metagenomes, 16S rRNA gene amplicon libraries, and published MAGs from the Marcellus, Utica, Niobrara, Bakken/Three Forks, and Duvernay regions (Daly et al. 2016, Borton et al. 2018b, Wang et al. 2019, Zhong et al. 2019, Zhang et al. 2020). Additional 16S rRNA gene amplicon datasets from Antrim and Barnett were included only in taxonomic comparisons because raw metagenomic sequences were not available for those sites.

Only the two newly generated Sichuan Basin metagenomes were locally assembled and binned as described in the shotgun metagenomic sequencing and genome-assembly section. For the published North American datasets, analyses were performed according to data availability: raw metagenomic reads were used for EMIRGE-based 16S rRNA gene reconstruction where available, whereas publicly available assembled metagenomes and MAGs in IMG were used for the functional gene and MAG-based comparisons.

**Figure 1:**
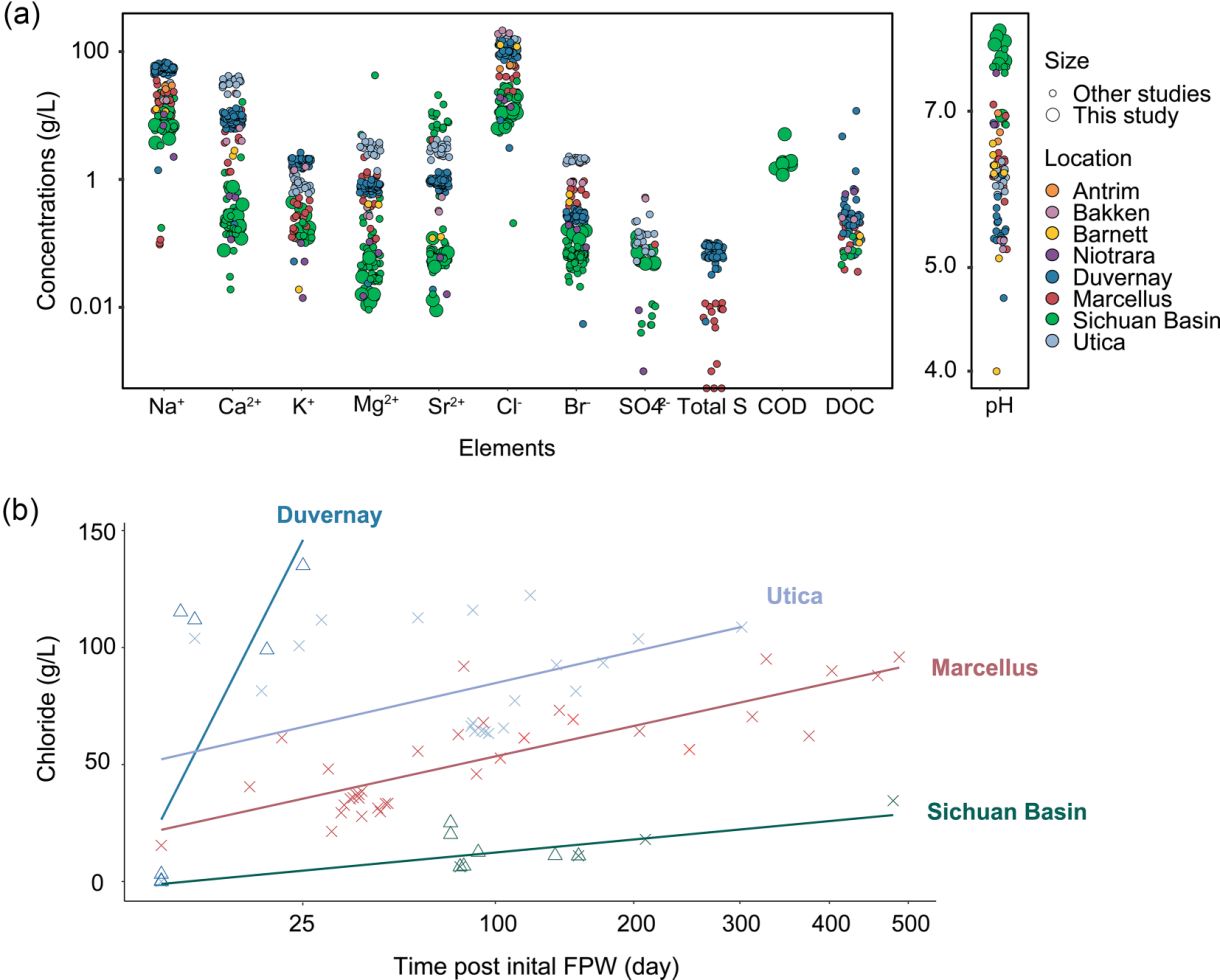
FPW chemistry among different hydraulic fracturing regions from unconventional oil and gas basins. (a) Overall flowback and produced water chemistry among unconventional oil and gas basins. Large circles represent data newly presented in this study, small circles represent data collected from previous studies (Davis et al. 2012, Wuchter et al. 2013, Cluff et al. 2014, Lester et al. 2015, Rosenblum et al. 2017, Ni et al. 2018, Wang et al. 2019, Blondes et al. 2020, Zhang et al. 2020) (for the raw data and source data, see Supplementary File 4). (b) Salinities and genomic sampling over time in FPW samples from the Sichuan Basin, Marcellus, Utica, and Duvernay. Trends show chloride concentrations and corresponding genomic samples, showing they well-covered the elevated salinity in the time series, representing stable flowback and produced water microbial communities in the late stage of the extraction process. “∆” represents 16S rRNA gene amplicon samples; “ × ” represents metagenomic samples.

We incorporated the newly generated geochemical data of FPW from the Sichuan Basin into the analyses based on the published geochemical data of FPW from the Sichuan Basin, Marcellus, Utica, Niobrara, Bakken, Antrim, Barnett, and Duvernay regions (Davis et al. 2012, Wuchter et al. 2013, Cluff et al. 2014, Lester et al. 2015, Rosenblum et al. 2017, Ni et al. 2018, Wang et al. 2019, Blondes et al. 2020, Zhang et al. 2020). For variables not newly measured in the present study, including the total sulfur values shown in Fig. 1, data were compiled directly from the corresponding source datasets listed in Supplementary File 4. We only included 16S rRNA gene amplicon data (see below) from three UOG wells of the Antrim Shale in Michigan, U.S. (Wuchter et al. 2013), and two UOG wells of Barnett Shale in Texas, U.S. (Davis et al. 2012), as raw metagenomic sequences from these two locations were not available. Bakken and Three Forks samples were combined as a single group for the 16S rRNA gene-based comparisons because both datasets were generated in the same study and represented closely related formations from the Williston Basin. Duvernay samples were included in the amplicon-based and geochemical comparisons, but not in the metagenome-derived 16S rRNA gene reconstruction or IMG-based functional comparisons because equivalent metagenomic datasets were not available for parallel processing in the present study.

#### Data processing for comparative analyses based on 16S rRNA gene sequences

For 16S rRNA gene sequencing data, the Illumina trimmed reads produced from the Sichuan Basin, Niobrara and Bakken were imported into QIIME2 v2025.7 (Bolyen et al. 2019). For shotgun metagenomic sequencing data, the 16S rRNA gene sequences from the Marcellus, Utica, and Sichuan Basins sample were reconstructed from trimmed Illumina reads using EMIRGE v 0.61.0 with 50 iterations (Miller et al. 2011). Then, EMIRGE-reconstructed 16S rRNA gene sequences were trimmed in silico to the V4 region corresponding to the 341F–806R amplicon target before import into QIIME2 (details in SI Text 9). Comparisons were interpreted cautiously. Both 16S rRNA gene amplicon data and 16S rRNA gene sequences reconstructed from shotgun metagenomes were classified using SILVA132 in the main comparative framework. Updated phylum-level nomenclature is used throughout the text and figures to reflect current taxonomy. The amplicon sequence variants (ASVs) labeled as “__” and “uncultured” are classified as unassigned. The comparability was assessed by analyzing sequencing depth from rarefaction curves for both 16S rRNA gene sequencing and metagenomic sequencing. For metagenome-derived taxonomic comparisons, all included metagenomic read datasets were processed using the same EMIRGE/QIIME2-based workflow. For the two newly analyzed Sichuan Basin metagenomes, sequencing depths were 5.96 and 6.27 Gb, respectively. Sequencing-depth comparability was assessed using rarefaction curves (Figure S1), and read-depth information for the included metagenomic datasets is provided in Table S1.

#### Comparative analyses of taxonomic profile and diversity analyses

Processed data produced from QIIME2 were imported to phyloseq package in R software (McMurdie and Holmes 2013) and taxonomic profiles were also visualized using R (v.4.1.3). Taxonomic compositions, including Venn diagrams, were analyzed using unrarefied datasets. For alpha-, beta-diversity, and network analyses, all sequences were rarefied to minimal counts (i.e. 2667 reads) using the rarefication function (for the rarefaction curves of the two datasets, see Figure S1) in the phyloseq package (McMurdie and Holmes 2013). Differences in microbial diversity (Shannon and Inverse Simpson diversity) and richness (Observed and Chao1 richness) among studied regions were measured using the diversity function implemented in the phyloseq package (McMurdie and Holmes 2013).

Co-occurrence network analyses were performed to identify recurrent associations among dominant microbial lineages and to examine whether methanogenic, sulfidogenic, and fermentative taxa formed conserved community modules across the analyzed basins.The network analyses were conducted using Bray–Curtis distance based on the microbial community compositions using the phyloseq package (McMurdie and Holmes 2013). The co-occurrence network analyses on the microbial community memberships following the method provided in Barberán et al. (2012). Spearman correlations were calculated using igraph v. 0.9.0 (Csárdi and Nepusz 2006, Bastian et al. 2009). Nodes and edges between genera (*P* > 0.01 and Spearman’s *ρ* < 0.8), and module parameters were calculated and visualized with the interactive platform gephi v.0.9.2. Additionally, Principal Coordinates Analysis (PCoA) using Bray–Curtis distance was used to measure the similarity of microbial community compositions among samples from different regions (Faith et al. 1987).

#### Comparative analyses of functional genes

Functional gene analyses of two new FPW samples from Sichuan Basin were done by using assembled metagenomic reads (Chen et al. 2017, 2019). The published assembled metagenomes from Marcellus and Utica in IMG were analyzed in parallel. The IMG Annotation Pipeline was applied to predict and translate coding sequences (CDS) and assign KEGG Orthology (KO) terms to the protein sequences. Annotation analyses of KEGG categories and pathways were also conducted (Chen et al. 2017, 2019). The relative abundance of genes classified in KEGG categories and pathways was compared, and the relative abundance of selected genes was compared. Bray–Curtis distance-based PCoA was used to measure the similarity of microbial community functions (based on KO) among samples (Faith et al. 1987). ANOVA analysis combined with TukeyHSD analysis was used to test whether the difference observed between groups was significant. *P* < 0.05 was regarded as a significant difference.

#### Phylogenetic and functional analyses of MAGs of FPW from the Sichuan Basin and MAGs previously published from North American Uog basins

A phylogenetic tree was of MAGs reconstructed using GtoTree based on Universal single-copy gene set (Hug et al. 2016, Lee and Ponty 2019). The maximum likelihood phylogenetic tree was calculated using FastTree v.2.1.11 with FastTree support values showing the relationships between medium to high quality MAGs of FPW in the Sichuan Basin to published MAGs from Marcellus and Utica (Daly et al. 2016, Borton et al. 2018b). Nodes with FastTree support values <70 were removed. MAGs were annotated using the DRAM (Distilled and Refined Annotation of Metabolism) annotation pipeline with default parameters to infer the functional capacity of the reconstructed MAGs (Shaffer et al. 2020). The presence of functional genes was measured with a set of identifiers using DRAM (Shaffer et al. 2020).

### Data availability

The 16S rRNA gene amplicon sequences and shotgun metagenomes from the Sichuan Basin analyzed in this study are publicly available. The 16S rRNA gene sequences are available under BioProject PRJNA628814 (SAMN14751469–SAMN14751477), and the two shotgun metagenomes are available in IMG under accession numbers 3300031260 and 3300031485. High-quality MAGs derived from the Sichuan Basin metagenomes are available under GOLD Study Gs0135899. These publicly available data are presented in Supplementary File 1.

## Results

### The geochemistry of FPW samples from the Sichuan Basin

The geochemistry of FPW provides insight into subsurface water-rock interaction and the broader geochemical context of the target reservoirs. The chemical profile of the newly collected FPW samples is presented in Table 3. For the eight new FPW samples from the Sichuan Basin, China, the pH of the FPW ranged from 6.93 to 8.33; COD varied from 1182 to 5109 mg L^−1^; and TDS varied from 10 800 to 47 002 mg L^−1^, and was mainly comprised of major cations such as Na^+^, Mg^2+^, Ca^2+^, and Sr^2+^. Cl^-^ concentrations ranged from 6302 to 25 147 mg L^−1^; SO_4_^2-^ concentrations ranged from 47.4 to 127.4 mg L^−1^, and NO_3_^-^ concentrations were in a similar range among these samples at approximately 75 mg L^−1^.

**Table 3:** Sampling and fluid chemistry.

Locations	Weiyuan							Zhaotong	
Wells	WY-3	WY-1	WY-2	WY-2	WY-2	WY-2	WY-3	ZT-1	ZT-2
Types	SW	FPW	FPW	FPW	FPW	FPW	FPW	FPW	FPW
Flowback time (day)	/	156	80	82	139	156	90	75	75
Sample index	1	2	3	4	5	6	7	8	9
pH	7.59	8.33	8.14	8.22	7.8	7.87	8.08	6.93	7.75
COD (mg L^−1^)	64	1696	1490	1876	1783	1946	5109	1765	1182
TDS (mg L^−1^)	1905	21 740	11 420	10 880	16 270	21 157	30 675	47 002	39 225
Cl^−^ (mg L^−1^)	25.05	10 987	6302	6609	11 111	11 013	12 509	25 147	20 142
SO_4_^2−^(mg L^−1^)	/	47.4	54	77	49.7	48.1	127.4	98.6	71.9
Br^−^ (mg L^−1^)	/	166.5	135.8	139.2	165.2	158.6	141.6	147.8	116.2
Na^+^ (mg L^−1^)	30	6416	4332	3753	6890	6714	7174	12 412	10 874
K^+^ (mg L^−1^)	2	168	122	130	168	177	275	443	341
Ca^2+^ (mg L^−1^)	57	265	78	119	278	273	188	769	406
Mg^2+^ (mg L^−1^)	3	15	11	12	16	16	30	68	60
Sr^2+^ (mg L^−1^)	1	55	9	13	56	54	43	121	72
NO_3_^−^ (mg L^−1^)	/	75	75	75	75	76	/	/	/

SW: source water; FPW: flowback and produced water; for samples undergoing 16S rRNA gene analyses and metagenomic analyses and without for samples exclusively undergoing 16S rRNA gene analyses.

FPW salinities in the Sichuan Basin, as indicated by various ion concentrations, were notably lower than those observed in most HF regions in North America, except Niobrara which had a similar low salinity level (Fig. 1a). The pH values of Sichuan Basin samples were alkaline, in contrast to the acidic conditions observed for samples from the North American UOG basins. Sulfur and carbon concentrations exhibited similar ranges across all regions. Figure 1b illustrates changes in salinity (measured by chloride concentrations) over time in the Sichuan Basin, Marcellus, Utica, and Duvernay, indicating substantial differences in terminal salinities. FPW samples from the Sichuan Basin consistently exhibited lower salinity compared to the other three locations at similar flowback time points.

### Taxonomic compositions

**Figure 2:**
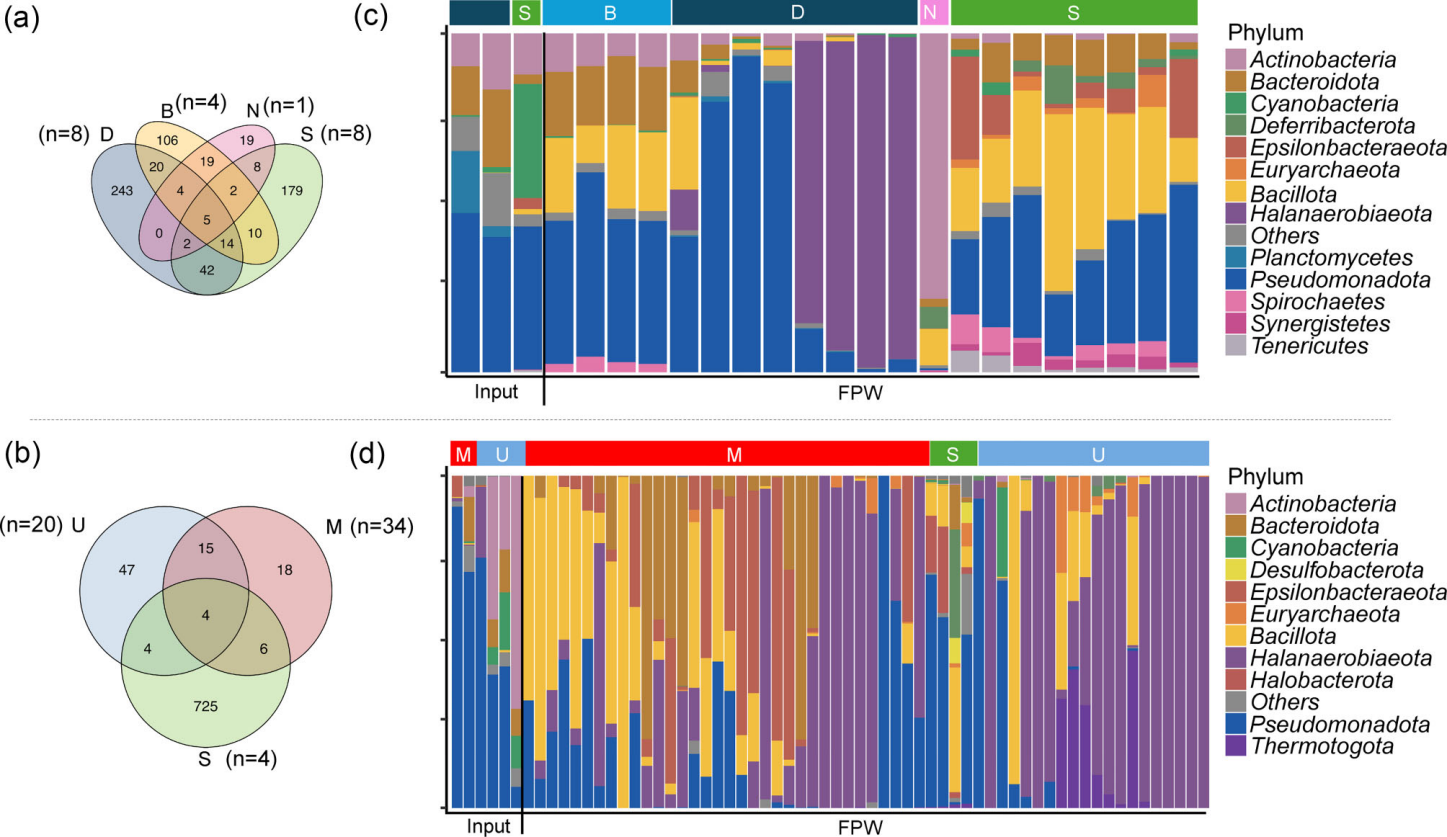
Taxonomic compositions for samples collected from six unconventional oil and gas plays in China and North America. Venn diagrams show genus-level distributions in FPW across hydraulic fracturing regions based on (a) 16S rRNA gene amplicon data and (b) metagenomic data. Taxonomic compositions of microbial communities at the phylum level in FPW and input fluids from the same hydraulic fracturing regions are shown based on (c) amplicon data and (d) metagenomic data. The same phylum color scheme is used in panels c and d, and phylum names follow current taxonomy. Samples within each basin in panels c and d are ordered by increasing time post initial FPW where such information was available. Taxa lacking genus-level classification were excluded from the Venn-diagram overlap analysis. For labels, S denotes Sichuan Basin, M denotes Marcellus, U denotes Utica, B denotes Bakken, N denotes Niobrara, and D denotes Duvernay.

For newly collected FPW samples in this study, an unrarefied dataset of eight 16S rRNA gene amplicon libraries contained a total of 262 ASVs (72 ASVs are unassigned genera), and 539 ASVs (235 ASVs are unassigned genera) were identified from two metagenomes. For assigned ASVs, the most abundant microbial phyla (average 2.2%–44.7%) in Sichuan Basin FPW samples were *Pseudomonadota, Bacillota, Deferribacterota, Bacteroidota, Desulfobacterota*, and *Euryarchaeota* based on the metagenomic data. At the genus level, *Thermovirga, Marinobacterium*, and *Desulfomicrobium* were abundant in both amplicon-based (average 2.0%–2.9%) and metagenome-based (average 1.4%–2.0%) datasets. The methanogens *Methanothermobacter* and *Methanolobus* were abundant Archaea in both amplicon-based (average 0.9%–1.0%) and metagenome-based (average 1.8%–2.6%) datasets. Among a significant proportion of unassigned ASVs, those belonging to *Deferribacteraceae* and *Rhodobacteraceae* families were notably enriched within the microbial communities. These taxonomic patterns are summarized in the genus-level Venn diagrams and phylum-level composition profiles shown in Fig. 2.

For the meta-analyses, the Venn diagram showed that most of the genera were unique to each location while fewer genera were present across HF regions (Fig. 2a and b). The dramatically different patterns of taxonomic compositions among different studied areas are also shown in the taxonomic profiles (Fig. 2c and d). For example, the metagenome-based data showed that only four genera, including *Thermococcus, Pseudomonas, Acinetobacter*, and an unclassified phylum bacterium, were present in FPW across the Sichuan Basin, Marcellus, and Utica plays. Consistent with previous reports from Marcellus and Utica systems, late-stage halophilic and thiosulfate-reducing Halanaerobium was not detected in the Sichuan Basin samples at comparable late-stage flowback times (Cluff et al. 2014, Daly et al. 2016, Booker et al. 2017).

### Alpha- and beta-diversity

**Figure 3:**
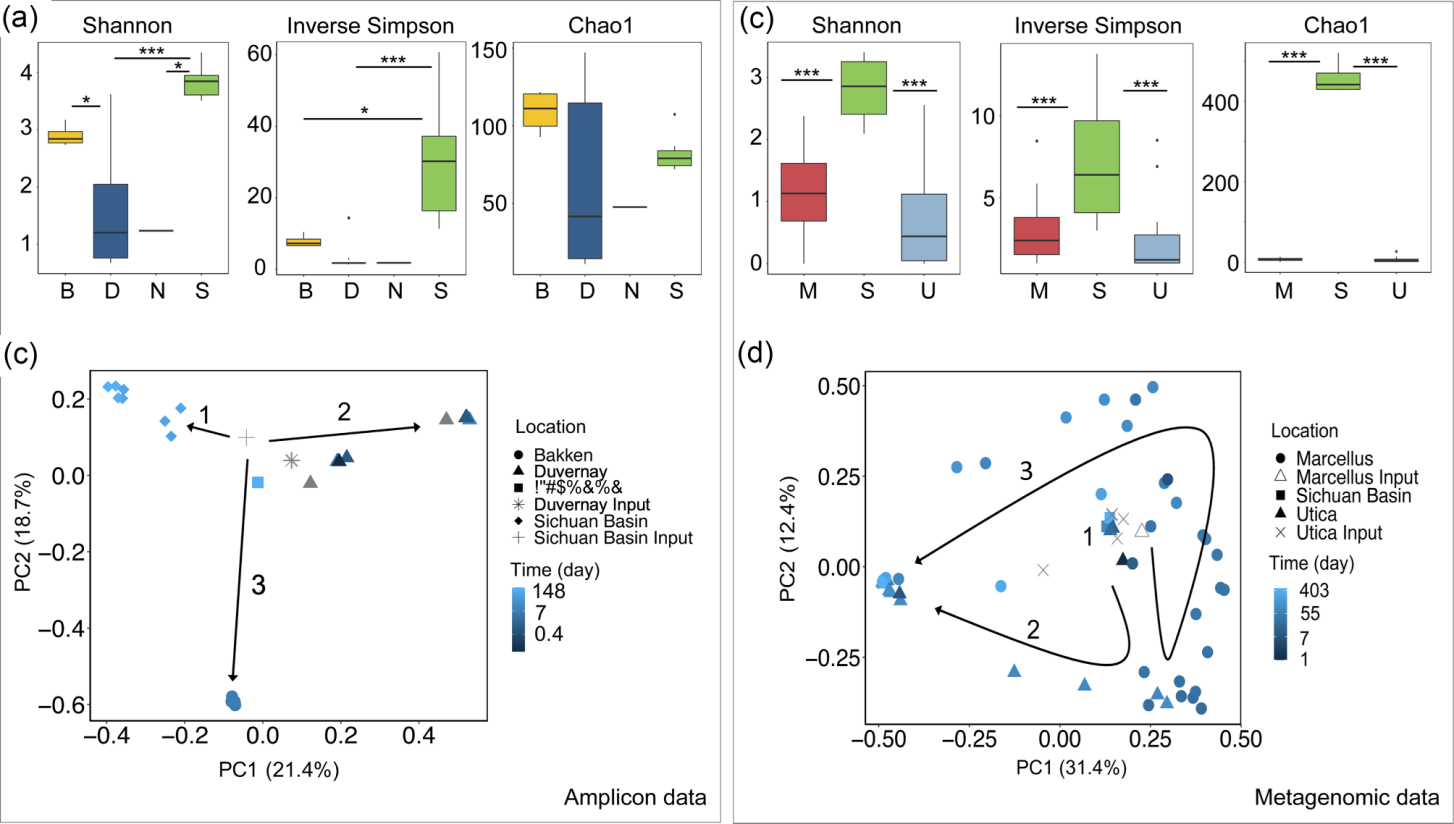
Diversity and succession trajectory of microbial communities from six unconventional plays in North America and China, including alpha diversity of (a) amplicon data and (b) metagenomic data for samples derived from different locations, and beta diversity of (c) amplicon data and (d) metagenomic data for samples derived from different locations, flowback time, and fluid types. In Fig. 3a and b, B denotes Bakken, D denotes Duvernay, N denotes Niobrara, S denotes Sichuan, M denotes Marcellus, and U denotes Utica. For Fig. 3c and d,sample timing after initial flowback is indicated in days in the figure legend. Numbers 1–3 represent the observed succession trajectories for microbial communities as FPW composition shifts from surface input water to late-stage flowback and produced water among studied regions.

Comparing microbial community diversity among studied areas, the Inverse Simpson and Shannon indices showed that the Sichuan Basin FPW samples were significantly more diverse than those from the Bakken, Niobrara, Marcellus, and Utica (Fig. 3a and b). The Chao 1 richness of the Sichuan Basin FPW samples was significantly higher than the Marcellus, and Utica. With regards to the diversity difference as a function of flowback time, the diversity was higher in late-stage Sichuan Basin FPW samples than in samples from Marcellus and the Utica at comparable flowback times (for additional statistical tests, see Supplementary Information).

The similarity distance of microbial community compositions in injected fluids collected from various regions were close (Fig. 3c and d). Thus, the injected fluid data could be used as reference points to measure community successions during well flowback. Using the amplicon data, three differing trajectories were identified for the Bakken, Duvernay, and Sichuan Basin. In the metagenome-based data, the trajectory of community composition changes from the Sichuan Basin FPW was minimal compared to that of the Utica and Marcellus. Regression analysis showed significant relationships between salinity-related measures and microbial diversity/richness across the analyzed datasets (Figure S3).

### Network analysis

**Figure 4:**
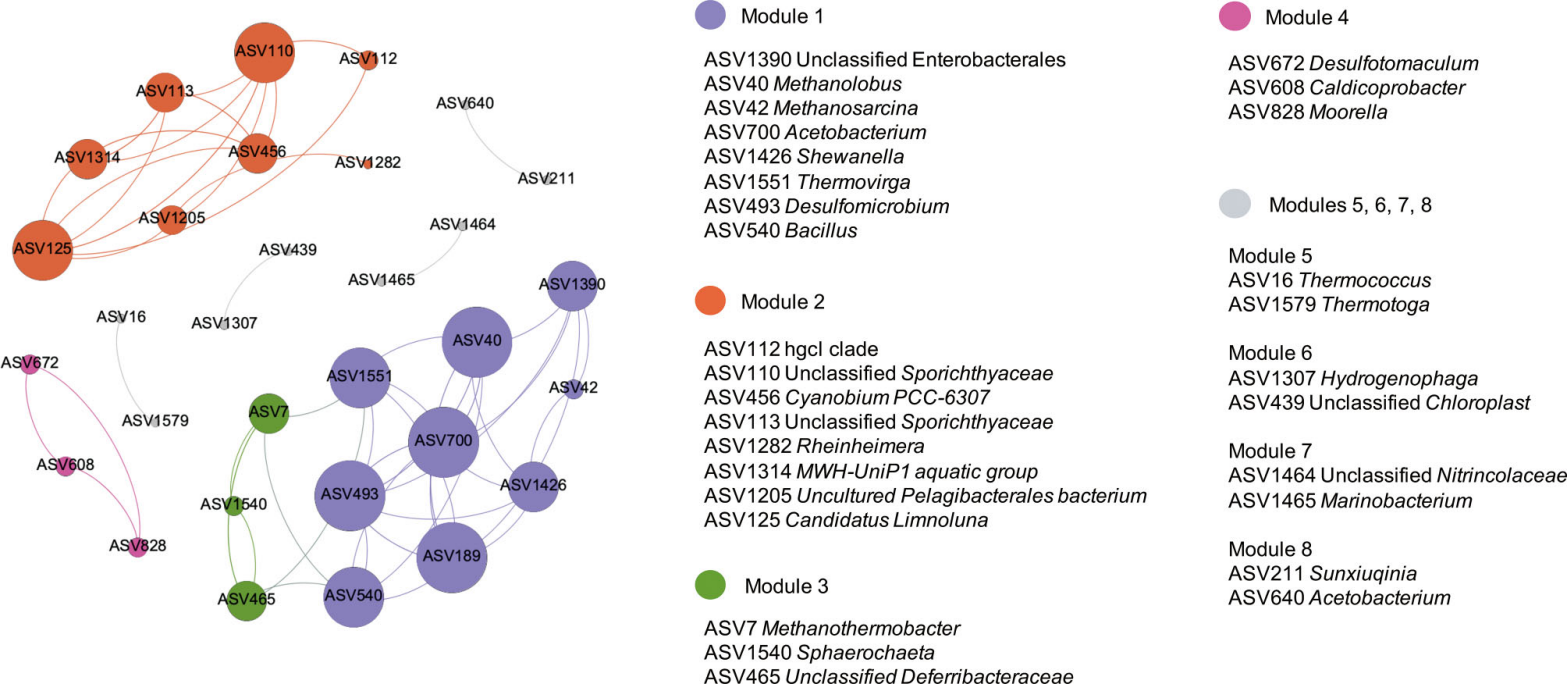
Co-occurrence network derived from the metagenome-derived taxonomic dataset. A connection indicates a strong (Spearman’s ρ > 0.8) and significant (*P* < 0.01) association. Node size represents average degree, and node color represents identified network modules. ASV labels and the corresponding taxonomic annotations for each module are shown to improve readability. Taxa and modules discussed in the text are labeled in the figure.

To identify recurrent associations among dominant microbial lineages across the analyzed basins, co-occurrence networks were constructed from both the metagenome-derived and 16S rRNA gene amplicon datasets. The metagenome-derived network is shown in Fig. 4, and the 16S rRNA gene amplicon-based network is provided in Figure S4.

In the metagenome-derived network, after filtering low-abundance microbial lineages (microbial genera comprising on average <0.1% of the community), 31 nodes and 53 edges with an average degree of 3.42 were identified. The network comprised eight separated modules of highly correlated microbial lineages, with nodes in Modules 1 and 2 showing higher numbers of connections than those in other modules. *Methanogenic lineages* were detected in Modules 1 and 3, including *Methanolobus* and *Methanosarcina* in Module 1 and *Methanothermobacter* in Module 3. These methanogens occurred in the same network with fermentative or organic-carbon-associated taxa such as *Acetobacterium, Bacillus*, and *Sphaerochaeta*, as well as sulfidogenic or sulfur-related taxa such as *Desulfomicrobium* and *Desulfotomaculum. Thermophilic lineages* were also observed in separate modules, including *Caldicoprobacter* and *Moorella* in Module 4 and *Thermococcus* and *Thermotoga* in Module 5.

For comparison, the 16S rRNA gene amplicon-based network contained 87 nodes and 405 edges with an average degree of 9.31 (Figure S4), indicating a more densely connected network structure. This network also showed the co-occurrence of sulfur-related bacteria and methanogens. In particular, microbial lineages such as *Methanothermobacter, Methanolobus, Desulfomicrobium, Dethiosulfatibacter*, and *Thermovirga* occurred in the core module of the network, whereas *Desulfuromonas* and *Methanobacterium* were detected in a separate module. Together, the two network analyses suggest recurrent associations among methanogenic, sulfidogenic, fermentative, and thermophilic micro-organisms in FPW microbial communities across the analyzed basins.

### Functional potentials

The functional potential of microbial communities in the flowback and produced water (FPW) was evaluated both from the metagenomic assembly and metagenome-assembled genomes (MAGs). Samples from the Sichuan Basin were analyzed in greater detail, as they were newly collected. The annotation statistics from IMG of the two newly sequenced metagenomes from the Sichuan Basin are presented in Table S2. We identified genes with both a significant response to subsurface conditions and high variability. For the gene-centric analyses of these two metagenomes, the counts of the dissimilatory sulfite reductase genes *dsrAB* were 16 and 26, and of the methanogenesis genes *mcrABG* (methyl-coenzyme M reductase) was 46 and 31. The relative abundances of the *dsrAB* and the *mcrABG* in the Sichuan Basin samples were significantly higher (*P* = <0.001–0.05) than for the Marcellus and the Utica basins. Relative abundances of functional genes associated with microbial persistence in hydrocarbon-bearing subsurface environments, including organic carbon degradation, flagellar assembly and cell motility, bacterial chemotaxis, salt resistance, and sporulation did not vary significantly between the different FPW sites (Figure S2).

**Figure 5:**
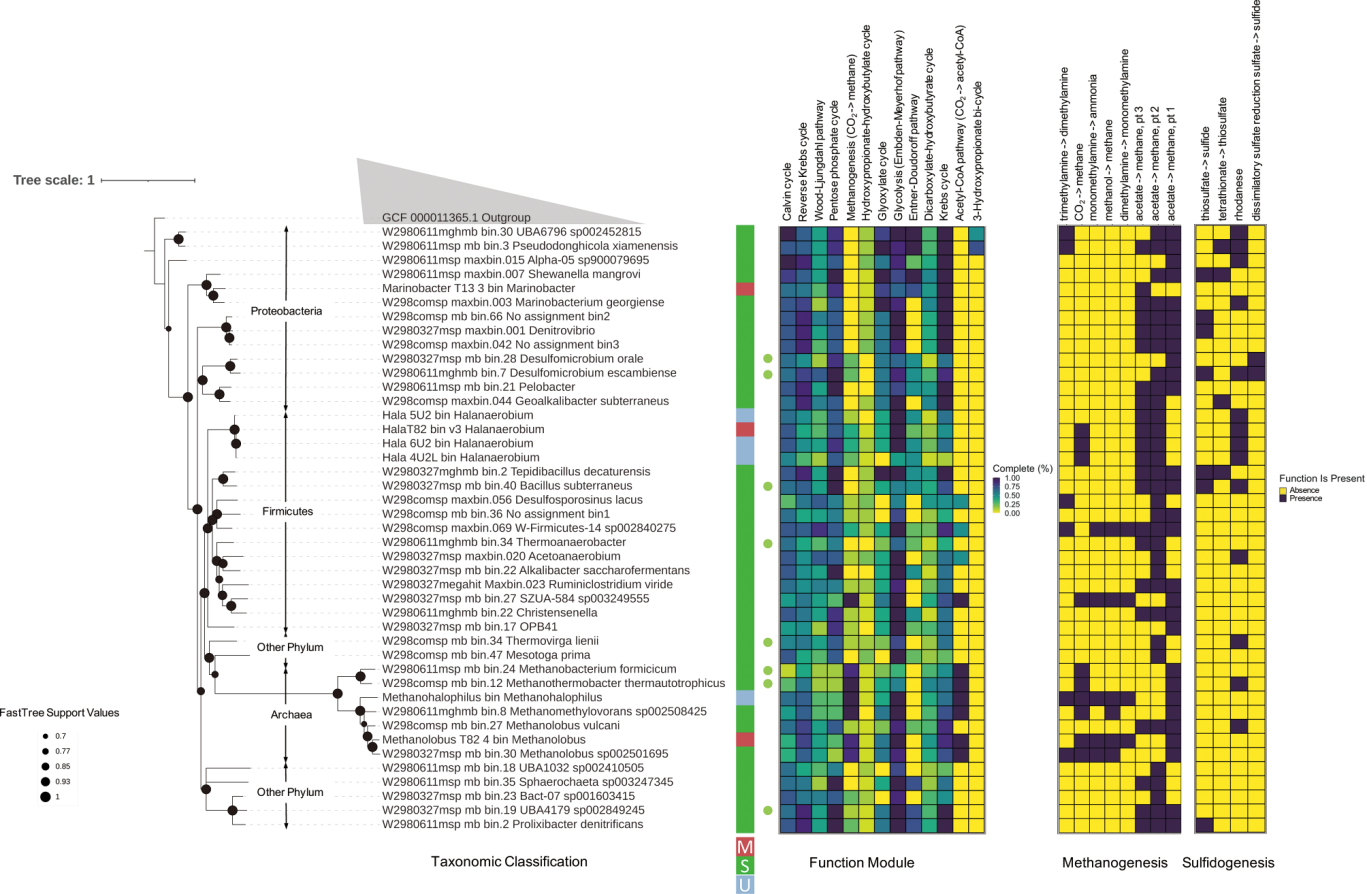
Phylogeny and predicted functional capacities of 36 metagenome-assembled genomes (MAGs) with reference MAGs derived from the Marcellus and Utica. Color bars represent MAGs derived from different locations, in which M denotes Marcellus, U denotes Utica, and S denotes Sichuan Basin. The green circles are manually refined MAGs with <100 contigs. The phylogenomic tree with support dots ≥ 70 was generated using GtoTree v. 1.7.05. The heatmap denotes the presence and absence of the functional genes related to sulfidogenesis and methanogenesis, which was annotated using DRAM v. 1.4.0.

A total of 36 medium-to-high quality metagenome-assembled genomes (MAGs) were generated from the two newly collected metagenomes from the Sichuan Basin (Supplementary File 3). The MAG-based phylogenetic relationships and predicted functional capacities are summarized in Fig. 5. Of these, 11 MAGs that met the predefined screening criteria (>90% completeness, <5% contamination, and <200 contigs) were selected for manual refinement (Table S3).

MAGs with relatively high abundance were taxonomically assigned to the thermophilic anaerobe *Thermovirga lienii* (representing 0.7% and 5.4% in the two samples, respectively) and the thermophilic methanogen *Methanothermobacter thermautotrophicus* (0.2% and 5.4%, respectively). This interpretation is also consistent with reported basin temperatures, with the Longmaxi Formation in the Sichuan Basin (59°C–61°C) being similar to the Marcellus and Utica (∼60°C) but lower than the Duvernay (>70°C) and Bakken (>122°C) (Daly et al. 2016, Gaspar et al. 2016, Chen et al. 2018). Two additional methanogen MAGs were classified as *Methanolobus* sp002501695 and *Methanolobus vulcani*. Although *Methanolobus* species have also been detected in the Marcellus, these exhibited only 77–78% ANI compared to their counterparts in the Sichuan Basin FPW. Other methanogen MAGs were identified as *Methanothermobacter formicicum* and *Methanothermobacter* sp00250842. All three major pathways of methanogenesis, including methylotrophic, hydrogenotrophic, and acetoclastic methanogenesis were detected in MAGs from the Sichuan Basin FPW. MAGs from the Marcellus and Utica regions exclusively contained genes associated with methylotrophic methanogenesis.

In the context of sulfur cycling, MAGs associated with sulfate-reducing bacteria (SRB) in the Sichuan Basin included *Desulfomicrobium orale* and *Desulfomicrobium escambiense*, and *dsrAB* genes were detected in these Sichuan Basin datasets. In contrast, published MAGs from the Marcellus and Utica included in our comparison did not contain SRB-affiliated MAGs or associated *dsrAB* genes.

## Discussion

### Location-specific taxonomic compositions and functional potentials

Microbial community composition differed among the analyzed basins (Fig. 2) and these differences were accompanied by differences in selected methanogenesis- and sulfur-related traits detected in the comparative analyses (Fig. 5). Despite that, the co-occurrence networks suggested recurrent associations among methanogenic, sulfidogenic, and fermentative micro-organisms in FPW across regions (Fig. 4 and Figure S4). Meta-analyses revealed highly diverse microbial communities in FPW from the Sichuan Basin, including *Thermovirga* and *Mesotoga* which are also commonly found in conventional hydrocarbon reservoirs (Nesbø et al. 2015, 2019). Among the diverse communities in the FPW from the Sichuan Basin, a considerable proportion of ASVs remained unassigned at the genus level under the analytical framework used in this study.

By adding more FPW samples from the Sichuan Basin for comparison, this study further characterized cross-basin differences in fractured subsurface microbial ecosystems. Specifically, the Sichuan Basin samples showed higher microbial diversity than several North American datasets, and the comparative analyses also indicated differences in selected methanogenesis- and sulfur-related traits among basins. These patterns are best interpreted in relation to basin-specific geological and geochemical context, rather than to salinity alone. For example, glycine betaine metabolism been linked to microbial adaptation under high salinity in Utica and Marcellus FPW was not detected in Sichuan Basin samples (Borton et al. 2018b, Jones et al. 2021, Daly et al. 2016 ).Other notable examples include regional differences in selected sulfur-related and methanogenesis-related traits. Consistent with previous studies, methanogenesis in the Marcellus and Utica appears to be restricted to methylotrophic pathways (Liu et al. 2012), with thiosulfate reduction mediated by the dominant fermentative bacterium *Halanaerobium* (Booker et al. 2017b).

### Salinity and temperature as filters of fractured subsurface microbial diversity

Fluid salinity is a strong environmental filter shaping microbial community composition and function (Oren 1994, 2001). Similar salinity-associated filtering has also been reported in hydraulically fractured shale systems, where highly saline late-stage FPW communities are often dominated by a narrower set of halotolerant taxa (Cluff et al. 2014, Daly et al. 2016, Borton et al. 2018b, Zhong et al. 2019). Our linear and logistic regression analyses revealed significant correlations between salinity and microbial diversity and richness (Figure S3). This study, together with previous studies (Zhong et al. 2019, 2020), identified a clear trend: Sichuan Basin samples with substantially lower salinity harbored fewer halophilic *Halanaerobium* but showed higher abundances of the methanogens *Methanothermobacter* and *Methanolobus*. For samples from the Marcellus, Utica, and Duvernay formations, the sequence-based data are consistent with persistence of methylotrophic methanogenesis under high-salinity conditions. This pattern may be related to the relatively higher free energy yield of methylotrophic methanogenesis compared to acetoclastic or hydrogenotrophic pathways, which may better support persistence under high-salinity conditions (Oren 2001).

Our analysis also highlighted the influence of subsurface temperatures on microbial community composition. Unlike the Marcellus and Utica formations, where salinity effects dominate, temperature played a more discernible role in shaping microbial populations in the Sichuan Basin. Notably, we identified abundant MAGs linked to thermophilic micro-organisms such as *Methanothermobacter thermautotrophicus* and *Thermovirga lienii*, both of which have optimal growth temperatures exceeding 50°C in cultured strains (Smith et al. 1997, Dahle and Birkeland 2006). Other relatives of thermophilic micro-organisms (*Thermotoga* and *Thermoanaerobacter*) were also detected in the Utica. The temperature of the Longmaxi Formation of the Sichuan Basin (59°C–61°C) is similar to that of the Marcellus and Utica Formations (∼60°C), which are considerably lower than the Duvernay (>70°C) and Bakken (>122°C) (Daly et al. 2016, Gaspar et al. 2016, Chen et al. 2018). The effects of elevated temperatures in the Duvernay and Bakken, combined with higher FPW salinity, have been discussed as reasons for low FPW biomass (Gaspar et al. 2016, Zhong et al. 2019).

### Implications of geochemical influences of petroleum geosystems

The geochemical conditions of petroleum geosystems, including source rock characteristics such as TOC, and kerogen type, play a primary role in controlling FPW chemistry. Differences in hydrocarbon composition among basins may further influence the types and availability of formation-derived substrates entering FPW and supporting microbial communities. At the basin/play level, the dominant hydrocarbon phase also differs across the systems compared here. The Wufeng–Longmaxi Formation in the Sichuan Basin is characterized by methane-dominated dry gas, whereas the Marcellus includes both dry-gas and more liquids-rich production domains depending on location within the play. The Utica spans oil-rich, wet-gas, and dry-gas windows, and the Duvernay similarly includes oil-, condensate/NGL-rich, and gas-prone intervals. In contrast, the Bakken/Three Forks system is predominantly oil-producing with associated gas. Although hydrocarbon composition was not directly measured in the present study, these basin-scale differences reported in previous literature likely influence the types and availability of formation-derived substrates entering FPW and therefore may contribute to cross-basin microbial differences (Feng et al. 2022,U.S. Energy Information Administration 2017a,b, National Energy Board 2017, U.S. Geological Survey 2013).Burial history and depositional setting further influence these conditions. Together, these factors shape the microbial ecosystems that develop in fractured subsurface environments. While detailed well-specific geological data are scarce, broader differences in depositional settings across basins provide indirect insights into microbial ecology. These differences help identify general patterns in microbial ecosystem formation (Table 1).

Salinity differences between the Sichuan Basin and major HF regions like the Marcellus, Utica, and Duvernay formations arise from distinct depositional environments. The Sichuan Basin, with isolated marine basins, contrasts with the open continental shelf or shallow marine settings of other regions. For example, the Upper Devonian Duvernay Formation was deposited as a shallow carbonate platform with localized reefs. The basin, located near the equator during deposition, likely experienced extensive evaporation that contributed to elevated salinity during burial and diagenesis and was ultimately associated with the production of high-salinity FPW in the present-day system. (Knapp et al. 2017, Harris et al. 2018). These geochemical conditions, shaped by source rock type and depositional setting, influence microbial ecology by determining available resources and environmental conditions for microbial life (Zhong et al. 2021b).

### Implications and future steps

Our results do not directly demonstrate biofouling, souring, or biocorrosion. Rather, they identify microbial taxa and selected functional traits that may be relevant to these microbiologically influenced processes in hydraulically fractured systems. For example, the detection of sulfate-reducing lineages and *dsrAB* genes in the Sichuan Basin is consistent with potential for sulfide generation, whereas cross-basin differences in methanogenic lineages and selected sulfur-related traits suggest that microbial operational risks may vary among sites depending on geological and geochemical context. Similar links between FPW microbiota and microbiologically influenced operational problems have been discussed in previous studies (Daly et al. 2016, Booker et al. 2017, Nixon et al. 2017). Future work should integrate microbiological observations with direct measurements of sulfide production, corrosion, and biofilm formation to better constrain site-specific operational implications.

### Limitations

This study has several limitations that should be considered when interpreting the cross-basin comparisons. First, the integrated dataset was compiled from different studies and therefore includes differences in sample sources, sequencing platforms, and data types. For example, some North American datasets were available as 16S rRNA gene amplicon libraries, whereas others were available only as shotgun metagenomic datasets. In the latter case, 16S rRNA gene sequences had to be reconstructed from metagenomes using EMIRGE rather than generated as matched amplicon datasets. Second, not all published datasets were available as raw reads suitable for complete reprocessing through a single uniform workflow. As a result, the newly generated Sichuan Basin metagenomes were locally assembled and binned, whereas some published functional and MAG-based comparisons relied on publicly available assemblies and MAGs in IMG. In addition, sample numbers differed among regions, with some groups represented by relatively few samples. This imbalance may influence regional diversity and richness comparisons and should be considered when interpreting cross-basin patterns.Because of this heterogeneity, direct one-to-one quantitative equivalence across all datasets should not be assumed. Accordingly, the results of this study are interpreted primarily as broad cross-basin ecological patterns rather than as fully pipeline-normalized comparisons.

## Supplementary Material

fiag070_Supplemental_Files
