# Supplementary material for: Geochemistry shapes microbial diversity and selected functional traits in flowback and produced waters from hydraulically fractured formations
Source: FEMS Microbiol Ecol. 2026 Jun 30;102(8):fiag070. doi: 10.1093/femsec/fiag070 (PMC13377646; doi:10.1093/femsec/fiag070)
Supplement: fiag070_Supplemental_Files [file fiag070_supplemental_files.zip › Zhong_SI_20260621.docx]

**Geochemistry shapes microbial diversity and selected functional traits in flowback and produced waters from hydraulically fractured formations**

Ya Deng^1^, Mikayla A. Borton ^2^, Camilla L. Nesbø ^3^, Malcolm D. Forster ^3^, Kurt O. Konhauser ^4^, Murray K. Gingras ^4^, Greg G. Goss ^3^, Kelly C. Wrighton ^2^, Brian D. Lanoil ^3^, Cheng Zhong ^1,4^*, Daniel S. Alessi ^4^*

1 College of Chemistry and Chemical Engineering, Southwest Petroleum University, Chengdu, Sichuan, China, 610500

2 College of Agricultural Science, Colorado State University, Fort Collins, USA

3 Department of Biological Sciences, University of Alberta, Edmonton, Canada

4 Department of Earth and Atmospheric Sciences, University of Alberta, Edmonton, Canada

Corresponding information *Tel: 1-512-232-0629, email: alessi@utexas.edu, chengzhong@swpu.edu.cn

Supplementary Information Contents

[Supplementary Information for Methods 3](#_Toc227265222)

[SI Text 1. pH measurements 3](#_Toc227265223)

[SI Text 2. COD measurements 3](#_Toc227265224)

[SI Text 3. Total dissolved solids 4](#_Toc227265225)

[SI Text 4. Cation analyses 4](#_Toc227265226)

[SI Text 5. Anion analyses 5](#_Toc227265227)

[SI Text 6. Cleaning, trimming, and checking quality of raw shotgun metagenomes derived from Sichuan Basin FPW samples 6](#_Toc227265228)

[SI Text 7. Manual refinement for MAG derived from Sichuan Basin FPW samples 6](#_Toc227265229)

[SI Text 8. Data distribution for FPW sample collected from North America 6](#_Toc227265230)

[SI Text 9. Extracting 16S rRNA gene from metagenomes 7](#_Toc227265231)

[Supplementary Tables 14](#_Toc227265232)

# Supplementary Information for Methods

### SI Text 1. pH measurements

pH was measured using a pH meter (PHSJ-3F, Shanghai INESA Scientific Instrument Co., Ltd., Shanghai , China) after calibration with standard buffer solutions according to the manufacturer’s instructions.

### SI Text 2. COD measurements

COD was determined using the dichromate reflux method following the standard protocol of the Ministry of Ecology and Environment of the People’s Republic of China (2017).

According to the formula, ρ (mg/L):

$$\rho=\frac{C\times(V_{0}-V_{1})\times8000}{V_{2}}\times f$$

*C*—Standard solution concentration of ammonium ferrous sulfate, mol/L;

*V_0_—*Volume of standard solution of ammonium ferrous sulfate consumed in blank test, mL;

*V_1_*—Volume of standard solution of ferrous ammonium sulfate consumed in water sample determination, mL;

*V_2_*—Volume of water sample taken during heating and reflux, mL;

*f*—Sample dilution ratio;

*8000*—Molar mass of $\frac{1}{4}$O_2_ in mg/L.

To unify the indicator used to evaluate the organic contents in each shale formation, we converted the chemical oxygen demand (COD) into total organic carbon (TOC), based on a model of influent wastewater (COD = 49.2 + 3.00×TOC) (Dubber and Gray 2010). Since all the samples were prefiltered through a 0.3 µm filter, TOC is assumed equivalent to DOC for comparative analysis.

### SI Text 3. Total dissolved solids

TDS was determined using the gravimetric method following the standard protocol of the Ministry of Ecology and Environment of the People’s Republic of China (1999). Filtered water samples were evaporated to dryness and reweighed to calculate TDS.

TDS:

$$C=\frac{W-W_{0}}{V}\times{10}^{6}$$

C—Total dissolved solids content, mg/L;

W—Total weight of evaporating dish and residue, g;

W_0_—Weight of evaporating dish, g;

V—Volume of Water sample, mL.

### SI Text 4. Cation analyses

Major cations were measured using an Atomic Absorption Spectrophotometer. 10 mL of water samples were filtered using 0.45 μm pore organic microporous membranes and the filtered samples were injected into the instrument test tank. The absorption wavelengths of each ion (K^+^: 766.5nm; Mg^2+^: 285.2nm; Na^+^: 589nm; Ca^2+^: 422.7nm; Sr^2+^: 430.2nm) were selected using the SP-3500AA (4AT) test software. The energy was automatically adjusted in the instrument (the energy balance is 50%; if the energy was too low or too high, the lamp current was adjusted). Then, the air compressor, acetylene and water-sealed instrument were opened to start the test (including blank and standard sample determination). Sample and standard curve data were saved.

### SI Text 5. Anion analyses

Preparation of 1000 ppm standard solutions: F^-^ (0.2210 g NaF added to 100mL deionized water); SO_4_^2-^ (0.1480 g Na_2_SO_4_ added to 100 mL deionized water); NO_3_^-^ (0.1371 g NaNO_3_ added to 100 mL deionized water); Cl^-^ (0.1651g NaCl added to 100 mL deionized water). All standard solutions were stored in polyethylene bottles at 4ºC. Preparation of mixed standard solutions: take 0.5 mL F^-^ solution, 0.75 mL Cl^-^ solution, 2.5 mL NO_3_^-^ solution, and 2.5 mL SO_4_^2-^ solution, and add them into a 250 mL volumetric flask; bring the total volume to 250 mL with ultrapure water. 5 mL aliquots (the standard sample was injected directly, and the water samples were filtered using 0.45 μm pore size filter membranes) were used for Ion Chromatography analyses. Balance mode was selected to balance the baseline (Baseline noise: 10 Hz; Resolution ratio: 0.0047 nS/cm). Samples were automatedly loaded into the instruments and analyzed after the instrument signal was baselined for 0.5-1 h. Samples and standard solutions were processed, and results recorded using the Magic Net software implemented in Ion Chromatography.

### SI Text 6. Cleaning, trimming, and checking quality of raw shotgun metagenomes derived from Sichuan Basin FPW samples

Preprocessing of the raw data obtained from the Illumina HiSeq sequencing platform was done using Readfq (https://github.com/cjfields/readfq) to acquire clean data for subsequent analysis. Clean data were blasted to the host database using Bowtie 2 software v 2.2.4 to remove potential human DNA contamination (Karlsson *et al.* 2012, 2013; Langmead and Salzberg 2013). Reads were trimmed and checked for quality using Trimmomatic v. 0.39 (Bolger, Lohse and Usadel 2014).

### SI Text 7. Manual refinement for MAG derived from Sichuan Basin FPW samples

Bins used for functional annotation were visually inspected for contamination using the anvi-refine interface in Anvi’o (Eren *et al.* 2015). To remove contaminating sequences not detected in Anvi’o, bins were processed using refineM v 0.1.1 (Parks *et al.* 2017), which identifies incorrectly binned contigs based on sequence composition and gene taxonomy. Bins were then imported into Geneious v 7.0.6 for further rounds of assembly and refinement (Kearse *et al.* 2012). The quality of finished MAGs was verified using CheckM.

### SI Text 8. Data distribution for FPW sample collected from North America

The input water and FPW were collected from five UOG wells of the Utica and Marcellus Formations in Ohio (n=2), West Virginia (n=2), and Pennsylvania (n=1) (Daly *et al.* 2016; Borton *et al.* 2018); one UOG well of the Niobrara Formation located in Colorado (Wang *et al.* 2019); two UOG wells of the Bakken Formation and two UOG wells of Three Forks Formation in North Dakota (Wang *et al.* 2019); and two UOG wells of the Duvernay Formation in Alberta (Zhong *et al.* 2019a).

### SI Text 9. Extracting 16S rRNA gene from metagenomes

16S rRNA genes were extracted from metagenomes using EMIRGE v0.61.1. EMIRGE sequences were chimera checked before phylogenetic gene analyses. To improve comparability between EMIRGE-derived 16S rRNA gene data and 16S rRNA gene amplicon data, reconstructed EMIRGE 16S rRNA gene sequences were trimmed in silico to the V4 region corresponding to the 341F–806R amplicon target. The trimmed EMIRGE-derived sequences were then processed through an in-house pipeline to generate QIIME2 input files for metagenome-based taxonomic analyses. Because sequence reconstruction and primer-based amplicon sequencing may still differ in sequence recovery and abundance representation, comparisons between these two datasets were interpreted with caution. This pipeline uses the NormPrior value calculated by EMIRGE to generate seqs.fna files for QIIME2 with the number of EMIRGE sequences reflected by NormPrior abundance with each sample having 1,000,000 sequences. For instance, if the NormPrior was 90%, it would have 900,000 sequences in the seqs.fna file, and 90% relative abundance in the QIIME2 amplicon sequence variants (ASV) table. EMIRGE-derived seq.fna files were converted to a biom file. Subsequently, both the EMIRGE-derived biom file and seqs.fna were imported into QIIME2.

**Diversity comparison**

We conducted ANOVA test for biodiversity of the late-stage FPW between Sichuan Basin, Utica, and Marcellus. Overall, the biodiversity as calculated as Shannon diversity in FPW from the Sichuan Basin (3.51–4.37, 16S rRNA gene amplicon-based; 2.09–3.40 metagenome-based) was within the range derived from the Duvernay (0.02–3.67, 16S rRNA gene amplicon-based), and the Marcellus FPW (0–2.38, metagenome-based) and Utica (0–2.51, metagenome-based). We further conducted tests for FPW samples >50 flowback days to exclude the diversity variance in early flowback influenced by input water. Our results are further supported by the fact that the diversity (*p* < 0.001) and richness (*p* < 0.001) indices of Sichuan Basin FPW were significantly higher those of the Utica and Marcellus.

**Methanogenesis and sulfidogenesis of the bin related microbes in previous studies**

Consistently, methylotrophic methanogenesis gene complexes (e.g., *mtmBC*, *mtbABC*, *mtaBC*, *mttBC*) were detected in *Methanohalophilus euhalobius* but were absent in *Methanothermobacter thermautotrophicus* and *Methanobacterium fomicicum* (Evans *et al.* 2019). According to previous studies, relatives of the MAGs related to methanogens from the Sichuan Basin FPW have been previously characterized as being able to use hydrogen (i.e., *Methanothermobacter thermautotrophicus* and *Methanobacterium fomicicum*) (Örlygsson *et al.* 1996; Wasserfallen *et al.* 2000) and methyl-C1 compounds (i.e., *Methanolobus vulcani* and *Methanomethylovorans* *thermophila*) (Kadam and Boone 1995; Jiang *et al.* 2005). The functional discrepancy was also indicated in previous site-specific studies of each UOG basin (Daly *et al.* 2016; Borton *et al.* 2018; Zhang *et al.* 2020).

Our annotation was consistent with previous studies showing that sulfite-reducing genes *dsrAB* that are key to dissimilatory sulfate reduction were not detected in the predominant sulfidogenic bacteria *Halanaerobium* in FPW samples from the Utica and the Marcellus (Booker *et al.* 2017a; Lipus *et al.* 2017). A recent study showed that no sulfate-reducing bacteria were detected through culturing in FPW from the Marcellus (Cliffe *et al.* 2020).

**Salinity filtering for microbial community memberships**

The relative abundance of *Halanaerobium* increased in FPW samples with increasing salinity (Murali Mohan *et al.* 2013; Cluff *et al.* 2014; Daly *et al.* 2016; Zhong *et al.* 2019b). In the cluster of FPW samples dominated by *Halanaerobium* (> 75% of sequences) in Marcellus, Utica, and Duvernay, *Halanaerobium* becomes predominant when the salinity is >63,000 mg L^-1^ chloride, which is consistent with the optimal growth salinity of *Halanaerobium* strains isolated from oil and gas sites (i.e., 60,000–90,000 mg L^-1^ chloride) (Oren 2015; An, Shen and Voordouw 2017; Booker *et al.* 2017b). This higher salinity can be found in the Utica FPW samples at 96 days following the initial FPW.

According to previous studies (lacking raw sequences for quantitative analyses), microbial community compositions in FPW from the Antrim Shale were more similar to those from the Sichuan Basin and the Niobrara (Wuchter *et al.* 2013). Consistent with this result, FPW produced from a UOG well from the Barnett was shown to be dominated by Halanaerobiales and Clostridiales (Davis, Struchtemeyer, and Elshahed, 2012), which are similar to succession endpoints of the Utica, Marcellus, and Duvernay. Consistent with our focus regions, the taxonomic profiles from Antrim and Barnett retrieved from previous literature are well correlated to their salinity levels.

**Supplementary Figures**

Figure S1 Rarefaction curves for (a) 16S rRNA gene sequence dataset and (b) metagenomic dataset.

Figure S2 Overall functional consistency of the flowback and produced water microbiome across spatial scales. (A) Relative abundance of overall genes classified to KEGG pathways and (B) relative abundance of selected key genes related to methanogenesis, sulfidogenesis, hydrocarbon degradation, alcohol and aldehyde hydrogenase, and sporulation. a represents p < 0.05 for samples between Sichuan Basin and Marcellus; b represents p < 0.05 for samples between Utica and Marcellus, c represents p < 0.05 for samples between Utica input and Marcellus, d represents p < 0.05 for samples between Utica and Sichuan Basin, e represents p < 0.05 for samples between Utica input and Sichuan Basin, f represents p < 0.05 for samples between Utica input and Utica.

Figure S3 Correlation between diversity indexes (Shannon diversity, Inverse Simpson diversity, Observed richness, and Chao1 richness) and salinities using data from the Sichuan Basin, Duvernay, Marcellus, and Utica. The top lanes are based on 16S rRNA gene amplicon analyses; the bottom lanes are based on the metagenome analyses. Data from Niobrara (single data point) and Bakken were excluded in these correlations.


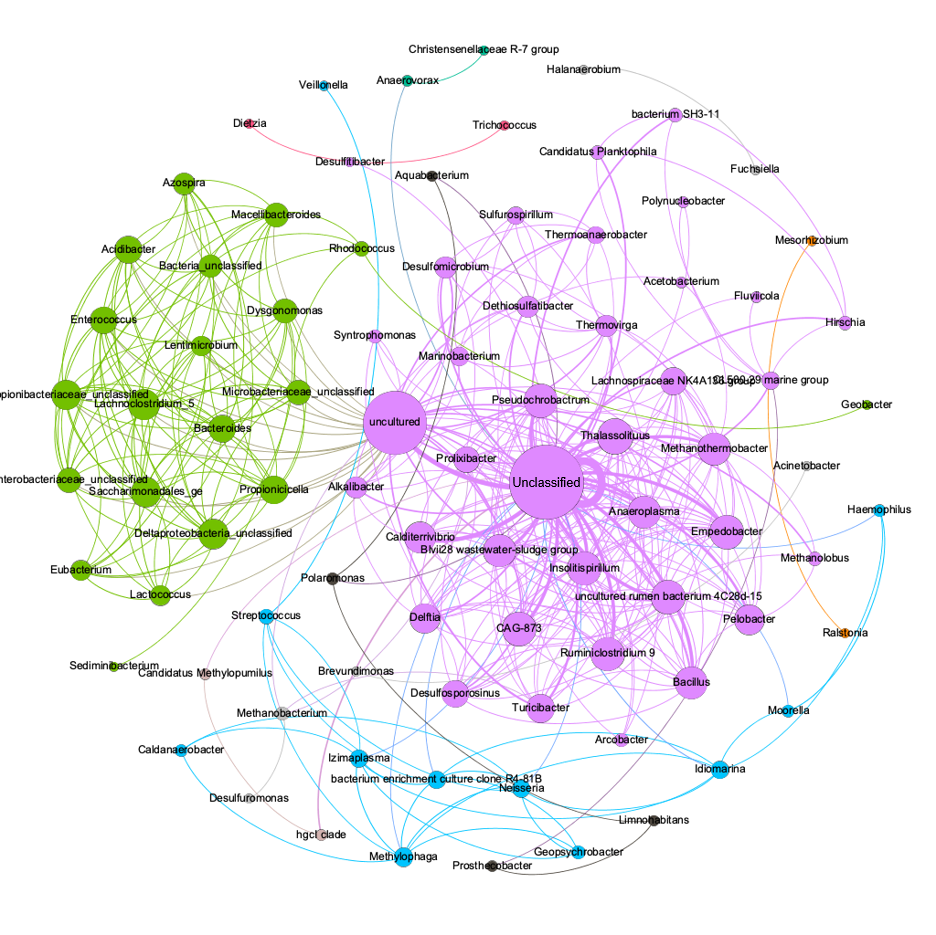


Figure S4. Co-occurrence network derived from the 16S rRNA gene amplicon dataset. A connection indicates a strong (Spearman’s ρ > 0.8) and significant (P < 0.01) association. Node size represents average degree, and node color represents identified network modules. Taxa discussed in the main text are labeled where possible to aid interpretation of the network structure.

Supplementary Tables

Table S1 List of the source and numbers of the 16S rRNA gene amplicon and shotgun metagenomic sequencing of flowback and produced water and fracturing input water for the comparative analyses in this study.

| Locations | Type | 16S rRNA gene amplicons | 16S rRNA gene extracts | Metagenomes (function) | Platform | Deposit | Flowback time (day) | | Reference | Accession numbers |
| --- | --- | --- | --- | --- | --- | --- | --- | --- | --- | --- |
| Sichuan Basin | FPW | 8 | 2 | 2 | Illumina | SRA/IMG | | 75-156 | This study | SAMN14751469- SAMN14751477/3300031260/3300031485 |
| Sichuan Basin | Input | 1 |  |  | Illumina | SRA | |  |  |  |
| Sichuan Basin | FPW |  | 2 |  | Illumina | SRA | | 210 and 480 | Zhang et al. (2020) | SAMN06216575 - SAMN06216581 |
| Duvernay | FPW | 8 |  |  | Illumina | SRA | | 0.04-18 | Zhong et al. (2019) | PRJNA407226 |
| Duvernay | Input | 2 |  |  | Illumina | SRA | |  |  |  |
| Marcellus | FPW |  | 34 | 16 | Illumina | SRA/IMG | | 7-488 | Borton et al. (2018) and Daly et al. (2016) | SAMN05172267 |
| Marcellus | Input |  | 1 |  | Illumina | SRA | |  |  | SAMN04432769 |
| Utica | FPW |  | 20 | 2 | Illumina | IMG | | 9-302 |  | SAMN06296050/ 06295989/ 06267276/ 06264872/ 07462262 |
| Utica | Input |  | 5 | 5 | Illumina | IMG | |  |  |  |
| Bakken | FPW | 2 |  |  | Roche 454 | SRA | | 9 | Wang et al. (2019) |  |
| Three Forks | FPW | 2 |  |  | Roche 454 | SRA | | 8 |  | SRP200534 |
| Niobrara | FPW | 1 |  |  | Roche 454 | SRA | | 140 |  |  |

Table S2 Basic sequencing annotation statistics

| Taxon_oid | 3300031260 | 3300031485 |
| --- | --- | --- |
| Domain | Microbiome | Microbiome |
| Sequencing Status | Finished | Finished |
| Sample | S.W2980611_MetaG | S.W2980327_MetaG |
| Location | Sichuan Basin | Sichuan Basin |
| Day post initial FPW | 156 | 80 |
| Sequencing Center | Beijing Novogene Bioinformatics Technology Co., Ltd | Beijing Novogene Bioinformatics Technology Co., Ltd |
| IMG Genome ID | 3300031260 | 3300031485 |
| Genome Size assembled | 136237194 | 200399001 |
| Gene Count assembled | 201139 | 307564 |
| Genome Bin Count assembled | 13 | 13 |
| GC assembled | 51.36552 | 44.725 |
| Coding Base Count % assembled | 99.73 | 99.75 |
| CDS Count assembled | 198802 | 304209 |
| CDS % assembled | 98.84 | 98.91 |
| RNA Count assembled | 2337 | 3355 |
| 16S rRNA Count assembled | 136 | 233 |
| RNA % assembled | 1.16 | 1.09 |
| Other RNA Count assembled | 0 | 0 |
| Unchar % | 0 | 0 |
| w/ Func Pred Count assembled | 142409 | 219509 |
| w/ Func Pred % assembled | 70.8 | 71.37 |
| w/o function prediction assembled | 56393 | 84700 |
| w/o function prediction % assembled | 28.04 | 27.54 |
| COG % assembled | 65.9 | 64.99 |
| KOG % | 0 | 0 |
| Pfam % assembled | 64.96 | 64.85 |
| TIGRfam % assembled | 0 | 0 |
| Enzyme % assembled | 26.73 | 26.82 |
| KEGG % assembled | 28.55 | 28.48 |
| Not KEGG % assembled | 70.29 | 70.43 |
| KO % assembled | 48.97 | 48.5 |
| Not KO % assembled | 49.86 | 50.41 |
| MetaCyc % assembled | 16.67 | 16.98 |
| Not MetaCyc % assembled | 82.17 | 81.93 |

Differences in assembled metagenome size may reflect sequencing depth, assembly recovery, and biological variation.

Table S3 Summary statistics of metagenome-assembled genomes (MAGs) reconstructed from FPW samples from the Sichuan Basin after manual refinements.

| MAGs | Taxa | Number of Contigs | N50, bp | GC content (%) | Complete-ness | Redun-dancy | Abundance (%) | | Contamina-tion (%) |
| --- | --- | --- | --- | --- | --- | --- | --- | --- | --- |
|  |  |  |  |  |  |  | W2_80 | W2_156 |  |
| W2980611mghmb_bin.8 | *Methanomethylovorans*  sp002508425 | 26 | 93587 | 37.71 | 81.58% | 0.00% |  | 0.16 | 0 |
| W2980611mghmb_bin.7 | *Desulfomicrobium*  *escambiense* | 72 | 72827 | 63.55 | 81.69% | 0.00% |  | 0.13 | 0 |
| W2980327msp_mb_bin.28 | *Desulfomicrobium*  *orale* | 18 | 118044 | 62.85 | 81.69% | 1.41% |  | 0.64 | 0 |
| W2980327msp_mb_bin.30 | *Methanolobus*  sp002501695 | 16 | 175801 | 49.31 | 93.42% | 0.00% |  | 1.07 | 0 |
| W2980327msp_mb_bin.40 | *Bacillus*  *subterraneus* | 32 | 123696 | 42.43 | 87.32% | 0.00% |  | 1.12 | 0 |
| W2980611msp_mb_bin.24 | *Methanobacterium*  *formicicum* | 35 | 33579 | 42.83 | 60.53% | 0.00% | 0.26 | | 1.35 |
| W2980611mghmb_bin.34 | *Thermoanaerobacter* | 50 | 26223 | 34.76 | 80.28% | 0.00% | 2.27 | | 0 |
| W2980327msp_mb_bin_19 | UBA4179 sp. | 53 | 62058 | 34.17 | 91.80% | 0.00% | 25.55 | 0.02 | 0.55 |
| W298comsp_mb_bin_27 | *Methanolobus vulcani* | 151 | 21560 | 40.64 | 92.81% | 0.00% | 0.01 | 0.52 | 0 |
| W2980327msp_mb_bin.34 | *Thermovirga lienii* | 40 | 75813 | 47.04 | 98.31% | 0.00% | 0.74 | 5.35 | 0 |
| W298comsp_mb_bin.12 | *Methanothermobacter thermautotrophicus* | 11 | 369779 | 49.60 | 100.00% | 0.00% | 0.16 | 5.38 | 0.25 |

W2_80, Weiyuan 2_80; W2_156, Weiyuan 2_156, Samples that have abundance for both wells are co-assembled genomes. N50 is a statistical measure of average length of a set of sequences.

**References**

An BA, Shen Y, Voordouw G. Control of Sulfide Production in High Salinity Bakken Shale Oil Reservoirs by Halophilic Bacteria Reducing Nitrate to Nitrite. *Front Microbiol* 2017;**8**, DOI: 10.3389/fmicb.2017.01164.

Bolger AM, Lohse M, Usadel B. Trimmomatic: A flexible Trimmer for Illumina Sequence Data. *Bioinformatics* 2014;**30**:2114–20.

Booker AE, Borton MA, Daly RA *et al.* Sulfide Generation by Dominant Halanaerobium Microorganisms in Hydraulically Fractured Shales. *mSphere* 2017a;**2**:e00257-17.

Borton MA, Hoyt DW, Roux S *et al.* Coupled Laboratory and Field Investigations Resolve Microbial Interactions that Underpin Persistence in Hydraulically Fractured Shales. *Proc Natl Acad Sci U S A* 2018;**115**:E6585–94.

Chaumeil P-A, Mussig AJ, Hugenholtz P *et al.* GTDB-Tk: A toolkit to classify genomes with the genome taxonomy database. *Bioinformatics* 2019;**36**:1925–7.

Chen F, Lu S, Ding X *et al.* Shale Gas Reservoir Characterization: A Typical Case in The Southeast Chongqing of Sichuan Basin, China. *PLoS One* 2018;**13**:1–16.

Cliffe L, Nixon SL, Daly RA *et al.* Identification of Persistent Sulfidogenic Bacteria in Shale Gas Produced Waters. *Front Microbiol* 2020;**11**:1–13.

Cluff MA, Hartsock A, Macrae JD *et al.* Temporal Changes in Microbial Ecology and Geochemistry in Produced Water from Hydraulically Fractured Marcellus Shale Gas Wells. *Environ Sci Technol* 2014;**48**:6508–17.

Daly RA, Borton MA, Wilkins MJ *et al.* Microbial Metabolisms in a 2.5-km-Deep Ecosystem Created by Hydraulic Fracturing in Shales. *Nat Microbiol* 2016;**1**:16146.

Davis JP, Struchtemeyer CG, Elshahed MS. Bacterial Communities Associated with Production Facilities of Two Newly Drilled Thermogenic Natural Gas Wells in the Barnett Shale (Texas, USA). *Microb Ecol* 2012;**64**:942–54.

Dubber D, Gray NF. Replacement of Chemical Oxygen Demand (COD) with Total Organic Carbon (TOC) for Monitoring Wastewater Treatment Performance to Minimize Disposal of Toxic Analytical Waste. *J Environ Sci Health A Tox Hazard Subst Environ Eng* 2010;**45**:1595–600.

Eren AM, Esen OC, Quince C *et al.* Anvi’o: An Advanced Analysis and Visualization Platformfor ’Omics Data. *PeerJ* 2015:PeerJ 3:e1319.

Evans PN, Boyd JA, Leu AO *et al.* An Evolving View of Methane Metabolism in The Archaea. *Nat Rev Microbiol* 2019;**17**:219–32.

Gaspar J, Davis D, Camacho C *et al.* Biogenic versus Thermogenic H2S Source Determination in Bakken Wells: Considerations for Biocide Application. *Environ Sci Technol Lett* 2016;**3**:127–32.

Jiang B, Parshina SN, van Doesburg W *et al.* Methanomethylovorans Thermophila sp. nov., A Thermophilic, Methylotrophic Methanogen from An Anaerobic Reactor Fed with Methanol. *Int J Syst Evol Microbiol* 2005;**55**:2465–70.

Jones AA, Pilloni G, Claypool JT *et al.* Evidence of Sporulation Capability of The Ubiquitous Oil Reservoir Microbe Halanaerobium Congolense. *Geomicrobiol J* 2020;**0**:1–11.

Kadam PC, Boone DR. Physiological Characterization and Emended Description of Methanolobus vulcani. *Int J Syst Bacteriol* 1995;**45**:400–2.

Kang DD, Froula J, Egan R *et al.* MetaBAT, An Efficient Tool for Accurately Reconstructing Single Genomes from Complex Microbial Communities. *PeerJ* 2015;**2015**:1–15.

Kanso S, Greene AC, Patel BKC. Bacillus subterraneus sp. nov., An Iron- and Manganese-Reducing Bacterium from A Deep Subsurface Australian Thermal Aquifer. *Int J Syst Evol Microbiol* 2002;**52**:869–74.

Karlsson FH, Fåk F, Nookaew I *et al.* Symptomatic atherosclerosis is associated with an altered gut metagenome. *Nat Commun* 2012, DOI: 10.1038/ncomms2266.

Karlsson FH, Tremaroli V, Nookaew I *et al.* Gut metagenome in European women with normal, impaired and diabetic glucose control. *Nature* 2013, DOI: 10.1038/nature12198.

Kearse M, Moir R, Wilson A *et al.* Geneious Basic: An Integrated and Extendable Desktop Software Platform for The Organization and Analysis of Sequence Data. *Bioinformatics* 2012;**28**:1647–9.

Langmead B, Salzberg S. Fast Gapped-read Alignment with Bowtie 2. *Nat Methods* 2013;**9**:357–9.

Lipus D, Vikram A, Ross D *et al.* Predominance and Metabolic Potential of Halanaerobium spp. in Produced Water from Hydraulically Fractured Marcellus Shale wells. *Appl Environ Microbiol* 2017;**83**, DOI: 10.1128/AEM.02659-16.

Ministry of Ecology and Environment of the People’s Republic of China. *HJ/T 51-1999 Water Quality: Determination of Total Salt-Gravimetric Method*., 1999.

Ministry of Ecology and Environment of the People’s Republic of China. *HJ 828-2017 Water Quality: Determination of the Chemical Oxygen Demand - Dichromate Method*., 2017.

Minoche AE, Dohm JC, Himmelbauer H. Evaluation of Genomic High-Throughput Sequencing Data Generated on Illumina HiSeq and Genome Analyzer Systems. *Genome Biol* 2011;**12**, DOI: 10.1186/gb-2011-12-11-r112.

Murali Mohan A, Hartsock A, Bibby KJ *et al.* Microbial Community Changes in Hydraulic Fracturing Fluids and Produced Water from Shale Gas Extraction. *Environ Sci Technol* 2013;**47**:13141–50.

Nurk S, Meleshko D, Korobeynikov A *et al.* MetaSPAdes: A New Versatile Metagenomic Assembler. *Genome Res* 2017;**27**:824–34.

Olm MR, Brown CT, Brooks B *et al.* DRep: A Tool for Fast and Accurate Genomic Comparisons That Enables Improved Genome Recovery from Metagenomes Through De-Replication. *ISME Journal* 2017;**11**:2864–8.

Örlygsson J, Krooneman J, Collins MD *et al.* Clostridium Acetireducens sp. nov., A Novel Amino Acid-Oxidizing, Acetate-Reducing Anaerobic Bacterium. *Int J Syst Bacteriol* 1996;**46**:454–9.

Parks DH, Imelfort M, Skennerton CT *et al.* CheckM: Assessing The Quality of Microbial Genomes Recovered from Isolates, Single Cells, and Metagenomes. *Genome Res* 2015;**25**:1043–55.

Parks DH, Rinke C, Chuvochina M *et al.* Recovery of nearly 8,000 metagenome-assembled genomes substantially expands the tree of life. *Nat Microbiol* 2017;**2**:1533–42.

Wang H, Lu L, Chen X *et al.* Geochemical and Microbial Characterizations of Flowback and Produced Water in Three Shale Oil and Gas Plays in The Central and Western United States. *Water Res* 2019;**164**:0043–1354.

Wasserfallen A, Nölling J, Pfister P *et al.* Phylogenetic Analysis of 18 Thermophilic Methanobacterium Isolates Supports the Proposals to Create A New Genus, Methanothermobacter gen. nov., and to Reclassify Several Isolates in Three Species, Methanothermobacter Thermautotrophicus comb. nov., Methano. *Int J Syst Evol Microbiol* 2000;**50**:43–53.

Wu Y-W, Tang Y-H, Tringe SG *et al.* MaxBin: An Automated Binning Method to Recover Individual Genomes from Metagenomes Using An Expectation Maximization Algorithm. *Microbiome* 2014;**2**:4904–9.

Wuchter C, Banning E, Mincer TJ *et al.* Microbial Diversity and Methanogenic Activity of Antrim Shale Formation Waters from Recently Fractured Wells. *Front Microbiol* 2013;**4**:1–14.

Zhang Y, Yu Z, Zhang Y *et al.* Regeneration of Unconventional Natural Gas by Methanogens Co-Existing with Sulfate-Reducing Prokaryotes in Deep Shale Wells in China. *Sci Rep* 2020;**10**:1–13.

Zhong C, Li J, Flynn SL *et al.* Temporal Changes in Microbial Community Composition and Geochemistry in Flowback and Produced Water from the Duvernay Formation. *ACS Earth Space Chem* 2019b;**3**:1047–57.
